# Supplementary material for: A new class of type VI secretion system effectors can carry two toxic domains and are recognized through the WHIX motif for export
Source: PLoS Biol. 2025 Mar 17;23(3):e3003053. doi: 10.1371/journal.pbio.3003053 (PMC12135965; doi:10.1371/journal.pbio.3003053)
Supplement: S1 Table — (DOCX) [file pbio.3003053.s001.docx]

**S1 Table. Bacterial strains used in this study.**

| **Strain name** | **Genotype** | **Comments** | **Source** |
| --- | --- | --- | --- |
| *Aeromonas jandaei* DSM 7311 | Wild-type | Used in competition assays, secretion assays, and for generating deletion strains. The strain is also named ATCC 49568 and CECT 4228 | DSMZ collection |
| *Aeromonas jandaei* ∆*tssB* | DSM 7311 ∆*we862_rs13035* | Used in competition and secretion assays | [1] |
| *Aeromonas jandaei* ∆*awe1* | DSM 7311 ∆*we862_rs20670* | Used in competition and secretion assays | This study |
| *Aeromonas jandaei* ∆*awe1*/∆*tssB* | DSM 7311 ∆*we862_rs13035* / ∆*we862_rs20670* | Used in competition and secretion assays | This study |
| *Aeromonas jandaei* ∆I-E-I | DSM 7311 ∆*we862_rs20675-we862_rs20665* | Used in competition assays | This study |
| *Aeromonas jandaei* ∆*tseI+imm* | DSM 7311  ∆*we862_rs13125-we862_rs13130* | Used in competition assays | This study |
| *Aeromonas jandaei* ∆*tle1+imm* | DSM 7311 ∆*we862_rs16925- we862_rs16920* | Used in competition assays | This study |
| *Aeromonas jandaei* ∆*duf3289+*Imm | DSM 7311 ∆*we862_rs09995*-downstream immunity (not annotated on NCBI) | Used in competition assays | This study |
| *Aeromonas jandaei* ∆*vgrG4* | DSM 7311 ∆*we862_20680* | Used in competition and secretion assays | This study |
| *Aeromonas jandaei* ∆*vgrG4*/∆*tssB* | DSM 7311 ∆*we862_20680* / ∆*we862_rs13035* | Used in competition and secretion assays | This study |
| *Aeromonas jandaei* *Aj*^effectorless^ | DSM 7311 ∆*we862_rs20670* / ∆*we862_rs16925* / ∆*we862_rs09995* / ∆*we862_rs13125* (nucleotides encoding amino acids 779-1509) | Used in competition assays | This study |
| *Escherichia coli* DH5α (λ-pir) | K-12 derivative laboratory strain containing λ-pir | Used for plasmid maintenance and cloning | Obtained from Eric V. Stabb |
| *Escherichia coli* BL21 (DE3) | Laboratory strain | Used as a prey in competition assays | Lab stocks |
| *Escherichia coli* MG1655 | Wild-type | Used in competition and toxicity assay | Lab stocks |
| *Escherichia coli* BTH101 | Δcya | BACTH reporter strain | Obtained from Graham Walker |

**References**

1. Jana B, Keppel K, Salomon D. Engineering a customizable antibacterial T6SS‐based platform in Vibrio natriegens. EMBO Rep. 2021;22: e53681. doi:10.15252/embr.202153681
