# Supplementary material for: A new class of type VI secretion system effectors can carry two toxic domains and are recognized through the WHIX motif for export
Source: PLoS Biol. 2025 Mar 17;23(3):e3003053. doi: 10.1371/journal.pbio.3003053 (PMC12135965; doi:10.1371/journal.pbio.3003053)
Supplement: S2 Table — (DOCX) [file pbio.3003053.s002.docx]

**S2 Table. Plasmids used in this study.**

| **Plasmid name** | **Description** | **Purpose** | **Source** |
| --- | --- | --- | --- |
| pBAD^K^/*Myc*-His | pBR322 ori-containing plasmid harboring a Kan^R^ cassette, *araC*, and an MCS following a P*bad* promoter. A Myc-His tag is encoded at the 5’ end of the MCS. | Used as an empty plasmid control | [1] |
| pAwe1 (also named pAwe1_Cyto_) | pBAD^K^/*Myc*-His plasmid containing *awe1*, in-frame with a C-terminal Myc-His tag | Used for arabinose-inducible expression of Awe1 | This study |
| pAwe1^1-699^ | pBAD^K^/*Myc*-His plasmid containing the region in *awe1* encoding Awe1^1-699^, in-frame with a C-terminal Myc-His tag | Used for arabinose-inducible expression of Awe1^1-699^ | This study |
| pAwe1^148-862^ | pBAD^K^/*Myc*-His plasmid containing the region in *awe1* encoding Awe1^148-862^, in-frame with a C-terminal Myc-His tag | Used for arabinose-inducible expression of Awe1^148-862^ | This study |
| pAwe1^148-699^ | pBAD^K^/*Myc*-His plasmid containing the region in *awe1* encoding Awe1^148-699^, in-frame with a C-terminal Myc-His tag | Used for arabinose-inducible expression of Awe1^148-699^ | This study |
| pGEX4T-1 | Lactose analog isopropyl  β-D-thiogalactoside  (IPTG)-inducible pGEX  plasmid harboring an  AmpR cassette | Used as a template for amplifying GST | GE Healthcare |
| pGST | pBAD^K^/*Myc*-His plasmid encoding GST (untagged) | Used for arabinose-inducible expression of GST | This study |
| pGST-Awe1^148-862^ | pBAD^K^/*Myc*-His plasmid encoding an N-terminal GST followed by a AAAGGG linker and Awe1^148-862^, in-frame with a C-terminal Myc-His tag | Used for arabinose-inducible expression of GST- Awe1^148-862^ | This study |
| pGST-Awe1^148-699^ | pBAD^K^/*Myc*-His plasmid encoding an N-terminal GST followed by a AAAGGG linker and Awe1^148-699^, in-frame with a C-terminal Myc-His tag | Used for arabinose-inducible expression of GST- Awe1^148-699^ | This study |
| pGST-Awe1^148-699^-GFP | pBAD^K^/*Myc*-His plasmid encoding an N-terminal GST followed by a AAAGGG linker, Awe1^148-699^, another AAAGGG linker, and sfGFP, in-frame with a C-terminal Myc-His tag | Used for arabinose-inducible expression of GST- Awe1^148-699^-GFP | This study |
| pGFP | pBAD^K^/*Myc*-His plasmid encoding superfolder GFP, in-frame with a C-terminal Myc-His tag | Used for arabinose-inducible expression of superfolder GFP | This study |
| psfGFP | pBAD33.1^F^ plasmid containing the CDS of sfGFP in-frame with a C-terminal FLAG tag | Used as template to amplify sfGFP | [2] |
| pAwe1^1-699^-GFP | pBAD^K^/*Myc*-His plasmid encoding an Awe1^1-699^, a AAAGGG linker, and sfGFP, in-frame with a C-terminal Myc-His tag | Used for arabinose-inducible expression of Awe1^1-699^-GFP | This study |
| pAwe1^148-699^-GFP | pBAD^K^/*Myc*-His plasmid encoding an Awe1^148-699^, a AAAGGG linker, and sfGFP, in-frame with a C-terminal Myc-His tag | Used for arabinose-inducible expression of Awe1^148-699^-GFP | This study |
| pPER5 | pBADK/Myc-His with a PelB signal peptide inserted at the 5' end of the MCS | Used for arabinose-inducible expression of proteins targeted to the periplasm | [3] |
| pAwe1_peri_ | pPER5 plasmid containing the CDS of Awe1, in-frame with an N-terminal PelB signal peptide and a C-terminal Myc-His tag | Used for arabinose-inducible expression of Awe1 in the periplasm | This study |
| pAwe1^1-147^_peri_ | pPER5 plasmid containing the CDS of Awe1^1-147^, in-frame with an N-terminal PelB signal peptide and a C-terminal Myc-His tag | Used for arabinose-inducible expression of Awe1^1-147^ in the periplasm | This study |
| pAwe1^700-862^_peri_ | pPER5 plasmid containing the CDS of Awe1^700-862^, in-frame with an N-terminal PelB signal peptide and a C-terminal Myc-His tag | Used for arabinose-inducible expression of Awe1^700-862^ in the periplasm | This study |
| pAwe1^148-699^_peri_ | pPER5 plasmid containing the CDS of Awe1^148-699^, in-frame with an N-terminal PelB signal peptide and a C-terminal Myc-His tag | Used for arabinose-inducible expression of Awe1^148-699^ in the periplasm | This study |
| pBAD33.1 | p15A ori-containing  plasmid carrying a  Cm^R^ gene, *araC*, and  an MCS following a  P*bad* promoter | Used as an empty plasmid control | Addgene |
| pBAD33.1^F^ | pBAD33.1 with a FLAG tag inserted at the 3' end of the MCS | Used for arabinose-inducible expression of proteins with a C-terminal FLAG tag | [4] |
| pBAD33.1^NF^ | pBAD33.1 with a FLAG tag inserted at the 5' end of the MCS | Used for arabinose-inducible expression of proteins with an N-terminal FLAG tag | This study |
| pTssB | pBAD33.1^F^ plasmid containing the CDS of TssB from *A. jandaei* DSM 7311 (untagged) | Used for arabinose-inducible expression of TssB | This study |
| pVgrG4 | pBAD33.1^NF^ plasmid containing the CDS of VgrG4 from *A. jandaei* DSM 7311, in-frame with an N-terminal FLAG tag | Used for arabinose-inducible expression of VgrG4 in *A. jandaei* | This study |
| pVgrG4^1-680^ | pBAD33.1^NF^ plasmid encoding VgrG4^1-680^ from *A. jandaei* DSM 7311, in-frame with an N-terminal FLAG tag | Used for arabinose-inducible expression of VgrG4^1-680^ in *A. jandaei* | This study |
| pAwiU | pBAD33.1^F^ plasmid containing the CDS of AwiU, in-frame with a C-terminal FLAG tag | Used for arabinose-inducible expression of AwiU | This study |
| pAwiD | pBAD33.1^F^ plasmid containing the CDS of AwiD, in frame with a C-terminal FLAG tag | Used for arabinose-inducible expression of AwiD | This study |
| pAwiU+ pAwiD | pBAD33.1^F^ plasmid containing the CDS of AwiU in the MCS, followed by another P*bad* promoter and the CDS of AwiD; Both AwiU and AwiD are in-frame with a C-terminal FLAG tag | Used for arabinose-inducible expression of AwiU and AwiD together | This study |
| pImm (TseI immunity) | pBAD33.1^F^ plasmid containing the CDS of the TseI immunity gene, in frame with a C-terminal FLAG tag | Used for arabinose-inducible expression of TseI immunity protein | This study |
| pImm (Tle1 immunity) | pBAD33.1^F^ plasmid containing the CDS of the Tle1 immunity gene, in frame with a C-terminal FLAG tag | Used for arabinose-inducible expression of Tle1 immunity protein | This study |
| pImm (DUF3289 immunity) | pBAD33.1^F^ plasmid containing the CDS of the DUF3289 immunity gene, in frame with a C-terminal FLAG tag | Used for arabinose-inducible expression of DUF3289 immunity protein | This study |
| pDM4 | a Cm^R^ and oriVR6K-containing suicide vector | Used as a template for constructing plasmids for gene deletions | [5] |
| pDM4:*tssB* | pDM4 containing 1 kb upstream and 1 kb downstream of the gene encoding TssB in *A. jandaei* DSM 7311 in its MCS | Used to delete *tssB* in *A. jandaei* DSM 7311 | [6] |
| pDM4:awe1 | pDM4 containing 1 kb upstream and 1 kb downstream of the gene encoding Awe1 in *A. jandaei* DSM 7311 in its MCS | Used to delete *awe1* in *A. jandaei* DSM 7311 | This study |
| pDM4:*awiU-awe1-awiD* | pDM4 containing 1 kb upstream of the gene encoding AwiU and 1 kb downstream of the gene encoding AwiD in *A. jandaei* DSM 7311 in its MCS | Used to delete *awiU*-*awe1-awiD* in *A. jandaei* DSM 7311 | This study |
| pDM4:*tle1* | pDM4 containing 1 kb upstream and 1 kb downstream of the gene encoding Tle1 in *A. jandaei* DSM 7311 in its MCS | Used to delete *tle1* in *A. jandaei* DSM 7311 | This study |
| pDM4:*tseI* | pDM4 containing 1 kb upstream and 1 kb downstream of the region encoding amino acids 779-1509 of TseI in *A. jandaei* DSM 7311 in its MCS | Used to delete *tseI* in *A. jandaei* DSM 7311 | This study |
| pDM4:*duf3289* | pDM4 containing 1 kb upstream and 1 kb downstream of the gene encoding the DUF3289-containing effector in *A. jandaei* DSM 7311 in its MCS | Used to delete *duf3289* in *A. jandaei* DSM 7311 | This study |
| pDM4:*tle1-imm* | pDM4 containing 1 kb upstream of the gene encoding the Tle1 effector and 1 kb downstream of the gene encoding its cognate immunity protein in *A. jandaei* DSM 7311 in its MCS | Used to delete *tle1* and its downstream immunity gene in *A. jandaei* DSM 7311 | This study |
| pDM4:*tseI-imm* | pDM4 containing 1 kb upstream of the gene encoding the TseI effector and 1 kb downstream of the gene encoding its cognate immunity protein in *A. jandaei* DSM 7311 in its MCS | Used to delete *tseI* and its downstream immunity gene in *A. jandaei* DSM 7311 | This study |
| pDM4:*duf3289-imm* | pDM4 containing 1 kb upstream of the gene encoding the DUF3289 effector and 1 kb downstream of the gene encoding its cognate immunity protein in *A. jandaei* DSM 7311 in its MCS | Used to delete *duf3289* and its downstream immunity gene in *A. jandaei* DSM 7311 | This study |
| pDM4:*vgrG4* | pDM4 containing 1 kb upstream and 1 kb downstream of the gene encoding VgrG4 in *A. jandaei* DSM 7311 in its MCS | Used to delete *vgrG4* in *A. jandaei* DSM 7311 | This study |
| pKT25 | A plasmid for BACTH assay containing the T25 fragment of the *сya* gene coding for adenylate cyclase, from *Bordetella pertussis* | Used as a template for constructing BACTH plasmids | Obtained from Graham Walker |
| pKT25:N-ter | pKT25 plasmid containing the CDS of Awe1^1-147^, in frame with the N-terminal T25 fragment of сya gene | Used in BACTH assays | This study |
| pKT25:C-ter | pKT25 plasmid containing the CDS of Awe1^148-862^, in frame with a N-terminal T25 fragment of сya gene | Used in BACTH assays | This study |
| pUT18C | A plasmid for BACTH assay containing the T18 fragment of the *сya* gene coding for adenylate cyclase from *Bordetella pertussis* | Used as a template for constructing BACTH plasmids | Obtained from Graham Walker |
| pUT18C:AwiU | pUT18C plasmid containing the CDS of AwiU, lacking the first 24 amino acids comprising its signal peptide, in frame with an N-terminal T18 fragment of the *сya* gene | Used in BACTH assays | This study |
| pUT18C:AwiD | pUT18C plasmid containing the CDS of AwiD, lacking the first 17 amino acids comprising its signal peptide, in frame with an N-terminal T18 fragment of the *сya* gene | Used in BACTH assays | This study |

**References**

1. Salomon D, Gonzalez H, Updegraff BL, Orth K. Vibrio parahaemolyticus Type VI secretion system 1 Is activated in marine conditions to target bacteria, and is differentially regulated from system 2. PLoS One. 2013;8: e61086. doi:10.1371/journal.pone.0061086

2. Dar Y, Jana B, Bosis E, Salomon D. A binary effector module secreted by a type VI secretion system. EMBO Rep. 2022;23: e53981. doi:10.15252/embr.202153981

3. Dar Y, Salomon D, Bosis E. The antibacterial and anti-eukaryotic Type VI secretion system MIX-effector repertoire in Vibrionaceae. Mar Drugs. 2018;16: 433. doi:10.1016/bs.ctdb.2015.10.001

4. Fridman CM, Keppel K, Gerlic M, Bosis E, Salomon D. A comparative genomics methodology reveals a widespread family of membrane-disrupting T6SS effectors. Nat Commun. 2020;11: 1085. doi:10.1038/s41467-020-14951-4

5. O’Toole R, Milton DL, Wolf-Watz H. Chemotactic motility is required for invasion of the host by the fish pathogen Vibrio anguillarum. Mol Microbiol. 1996;19: 625–637. doi:10.1046/j.1365-2958.1996.412927.x

6. Jana B, Keppel K, Salomon D. Engineering a customizable antibacterial T6SS‐based platform in Vibrio natriegens. EMBO Rep. 2021;22: e53681. doi:10.15252/embr.202153681
