# Supplementary figures and images for: A new class of type VI secretion system effectors can carry two toxic domains and are recognized through the WHIX motif for export
Source: PLoS Biol. 2025 Mar 17;23(3):e3003053. doi: 10.1371/journal.pbio.3003053 (PMC12135965; doi:10.1371/journal.pbio.3003053)

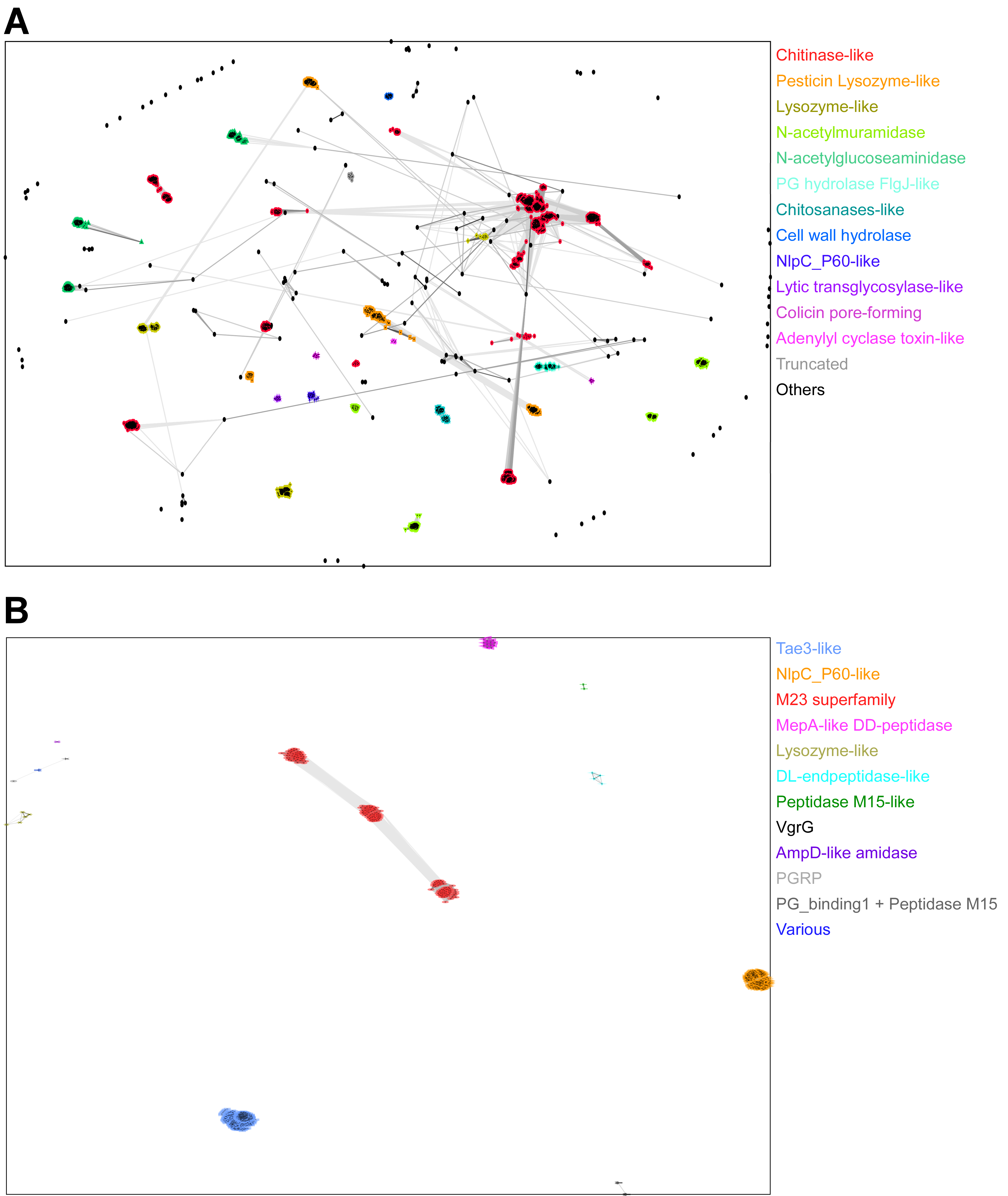

Supplement: S1 Fig — Sequences C-terminal (last 100 amino acids) (A) or N-terminal (B) to WHIX domains were clustered in two dimensions based on all-against-all sequence similarity, using the CLANS application, with nodes representing unique sequences and connecting lines representing the distances between sequences. The predicted activities or domains identified in each cluster are denoted and color-coded according to the nodes. In (A), black nodes (others) comprise clusters of <4 members, which were not analyzed. The data underlying this figure can be found in S2 File (A) and S3 File (B). (TIF) [file pbio.3003053.s003.tif]

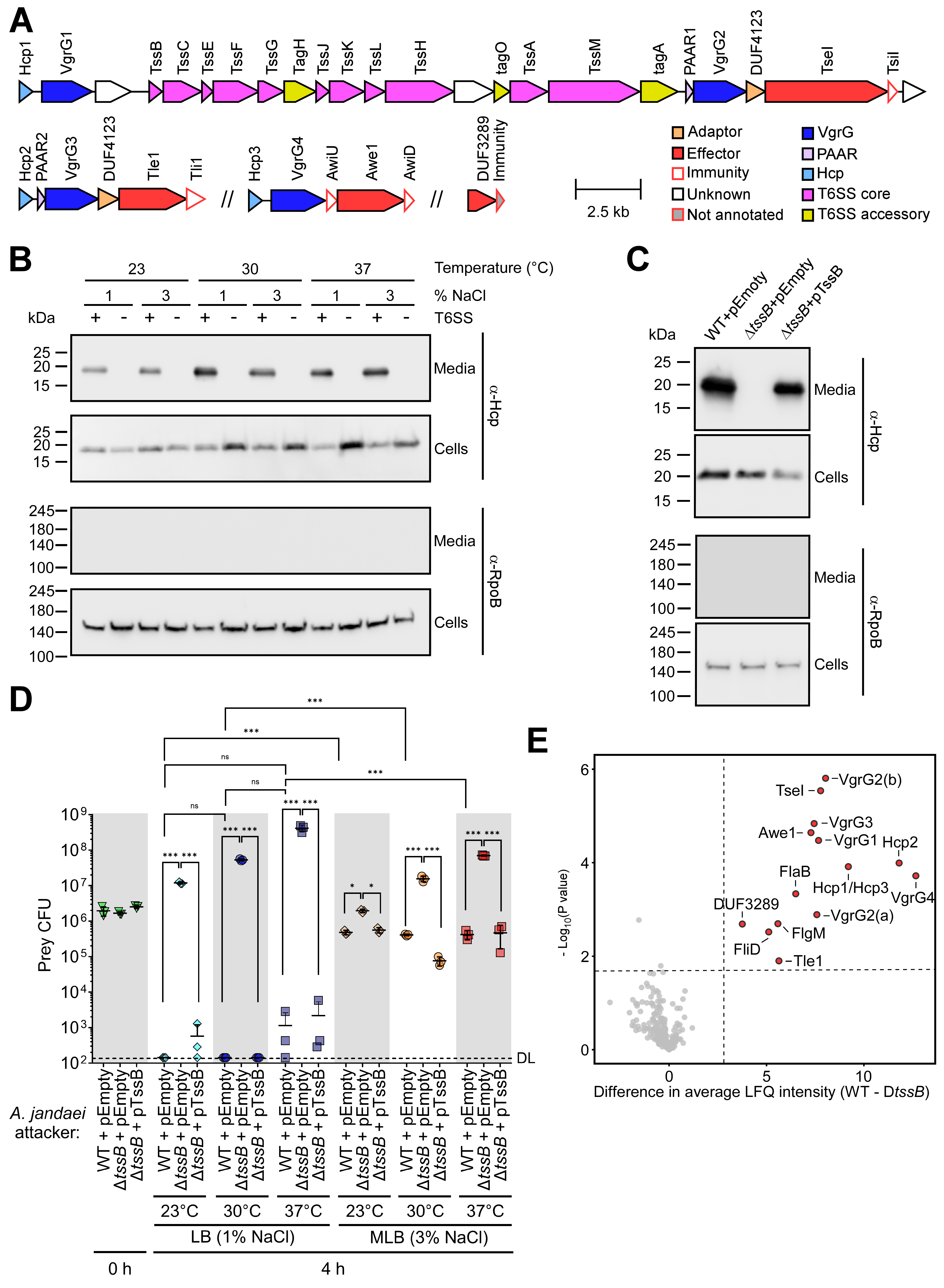

Supplement: S2 Fig — (A) Schematic representation of A. jandaei DSM 7311 T6SS gene cluster and auxiliary operons. Predicted protein activity is denoted above. (B and C) Expression (cells) and secretion (media) of Hcp from wild-type (WT; T6SS+) A. jandaei DSM 7311 and a T6SS− mutant strain (∆tssB) grown for 3 h at the indicated temperatures in media containing 1% or 3% (wt/vol) NaCl (LB or MLB, respectively). RNA polymerase beta subunit (RpoB) was used as a loading and lysis control. Results from a representative experiment out of at least three independent experiments are shown. In (C), bacterial strains contain either an empty plasmid (pEmpty) or a plasmid for the arabinose-inducible expression of tssB (pTssB), and the assay was performed at 30 °C in LB media supplemented with chloramphenicol and 0.1% (wt/vol) l-arabinose. (D) Viability counts (colony forming units; CFU) of E. coli MG1655 prey strains before (0 h) and after (4 h) co-incubation with the indicated A. jandaei DSM 7311 attacker strains containing an empty plasmid (pEmpty) or a plasmid for the arabinose-inducible expression of tssB (pTssB), on LB plates supplemented with 0.1% (wt/vol) l-arabinose at 30 °C. The statistical significance between samples was calculated using two-way ANOVA with Tukey multiple comparisons test on log-transformed data; ****P < 0.0001; *P = 0.0353 or 0.0362; ns, no statistical significance (P > 0.05); WT, wild-type; DL, the assay’s detection limit. Data are shown as the mean ± SD; n = 3. The data shown are a representative experiment out of at least three independent experiments. The data underlying this figure can be found in S4 Data. (E) Volcano plot summarizing the comparative analysis of proteins identified in the media of A. jandaei DSM 7311 WT and T6SS− (ΔtssB) strains, using label-free quantification (LFQ). The average LFQ signal intensity difference between the WT and T6SS− strains is plotted against the −Log10 of Student t-test P-values (n = 3 biological replicates). Proteins that were sig [file pbio.3003053.s004.tif]

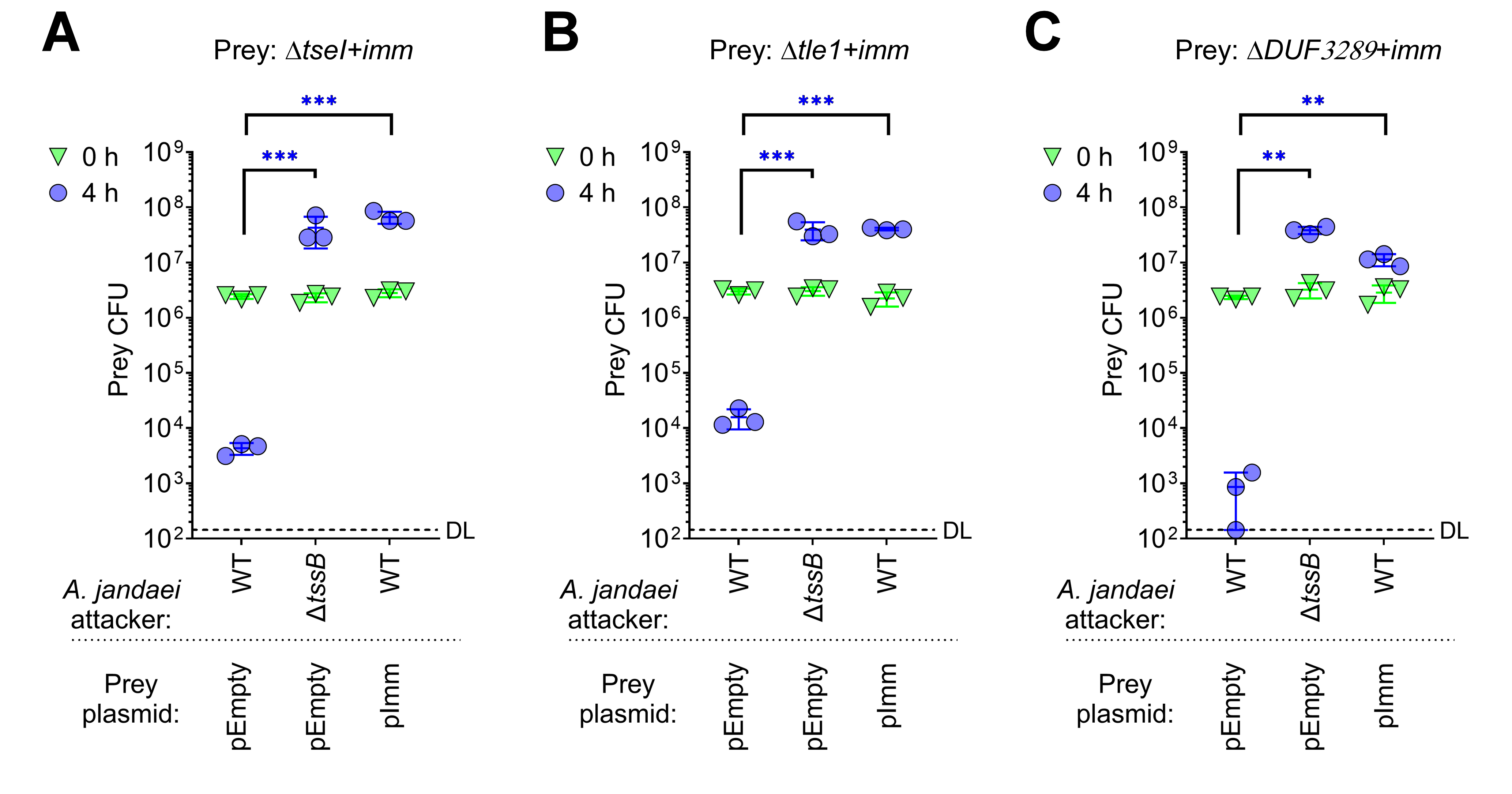

Supplement: S3 Fig — Viability counts (CFU) of A. jandaei DSM 7311 prey strains in which the genes encoding TseI (A), Tle1 (B), and DUF3289 (C) were deleted with their predicted downstream immunity genes. Prey strains contain an empty plasmid (pEmpty) or a plasmid for the arabinose-inducible expression of the predicted cognate immunity protein (pImm), before (0 h) and after (4 h) co-incubation with the indicated A. jandaei DSM 7311 attacker strains on LB plates supplemented with 0.05% (wt/vol) l-arabinose at 30 °C. The statistical significance between samples at the 4-h time point was calculated using an unpaired, two-tailed Student t-test on log-transformed data; ***P < 0.0001; **P < 0.0002; WT, wild-type; DL, the assay’s detection limit. Data are shown as the mean ± SD; n = 3. The data shown are a representative experiment out of at least three independent experiments. The data underlying this figure can be found in S5 Data. (TIF) [file pbio.3003053.s005.tif]

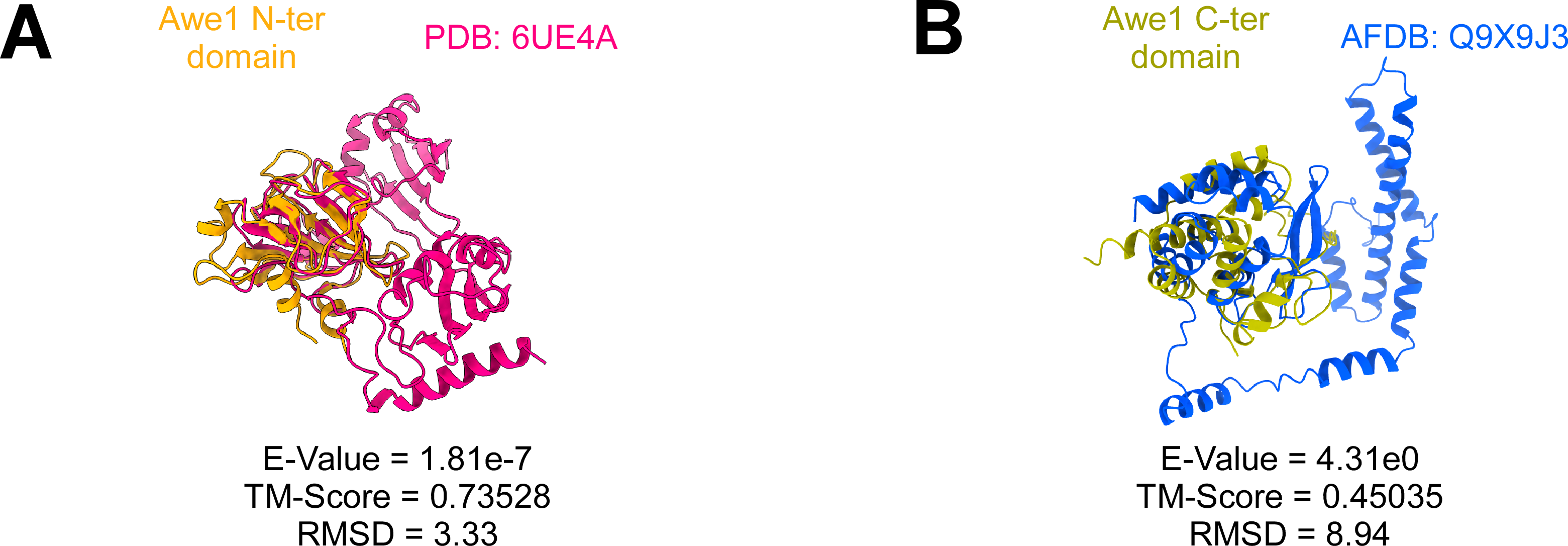

Supplement: S4 Fig — (A) Superimposed AlphaFold3 structure prediction of the N-terminal (N-ter) Awe1 domain (amino acids 1–147; colored orange) with Vibrio cholerae ShyA (PDB: 6UE4A; colored fiusha). (B) Superimposed AlphaFold3 structure prediction of the C-terminal (C-ter) Awe1 domain (amino acids 700–862; colored beige) with the AlphaFold structure prediction of Vibrio parahaemolyticus FlgJ (AlphaFold database [AFDB]: Q9X9J3; colored blue). The data underlying this figure can be found in S7 File. (TIF) [file pbio.3003053.s006.tif]

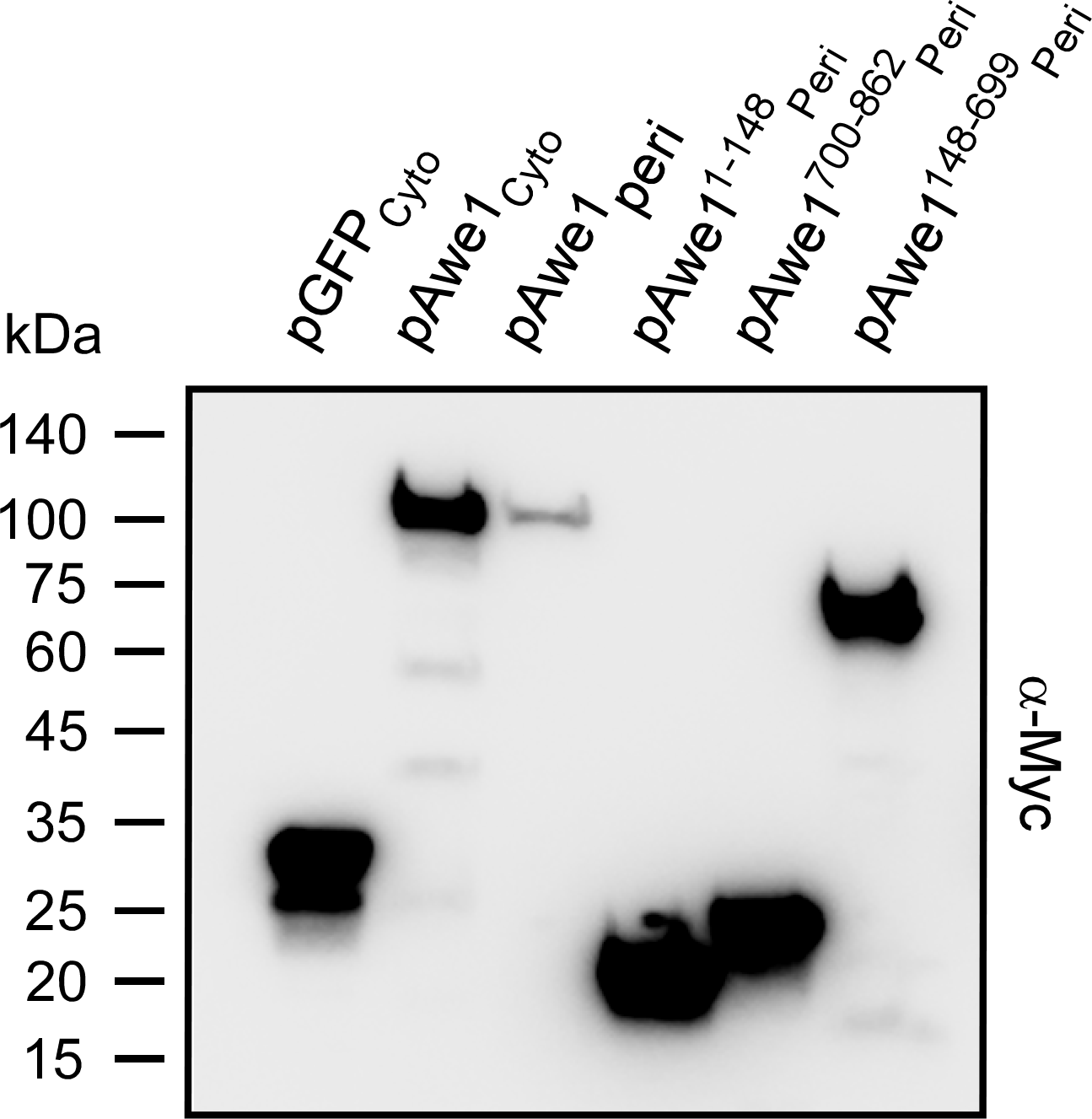

Supplement: S5 Fig — The expression of the indicated C-terminally Myc-tagged superfolder GFP and Awe1 forms expressed in the cytoplasm (Cyto) or periplasm (Peri) of Escherichia coli MG1655 from an arabinose-inducible plasmid. (TIF) [file pbio.3003053.s007.tif]

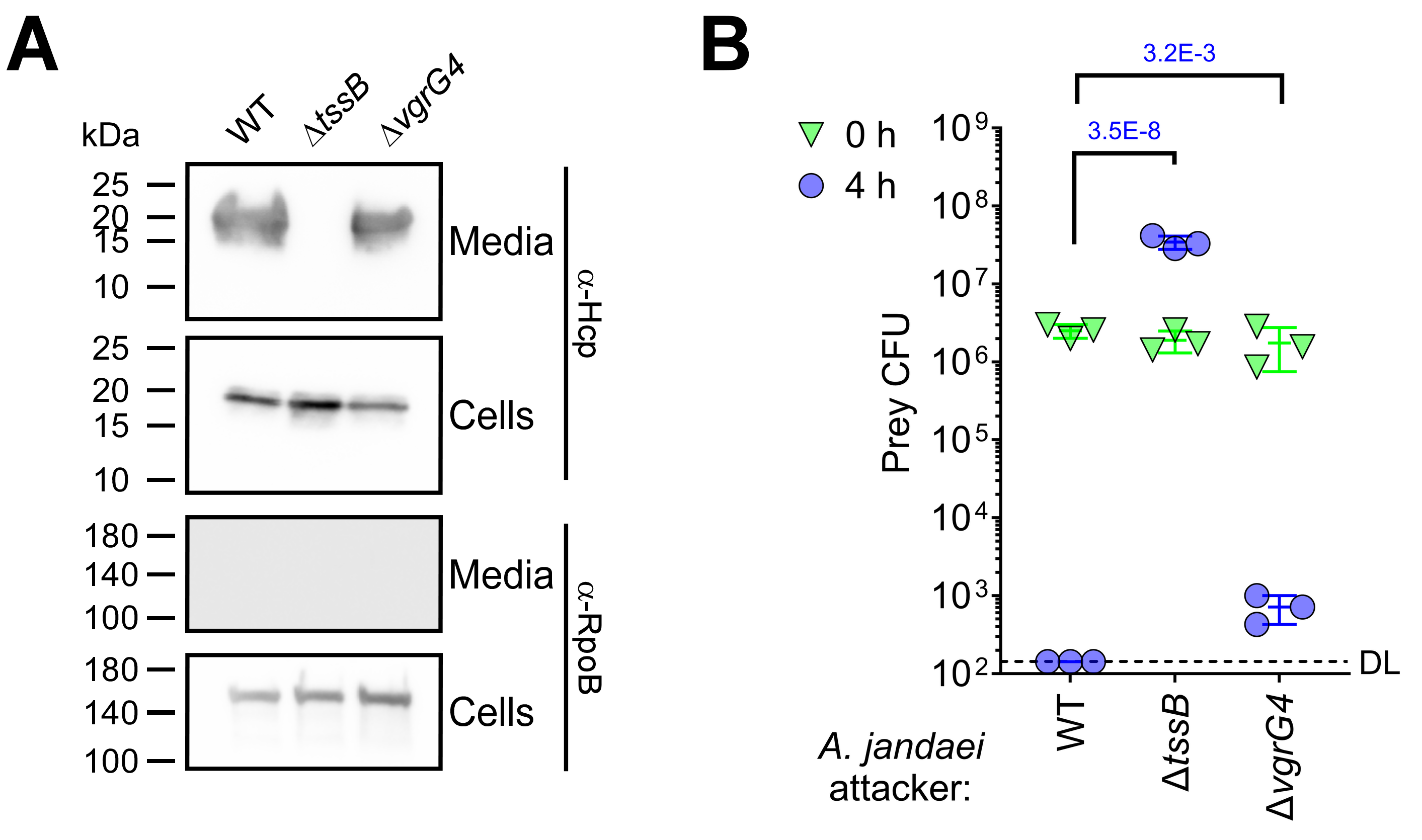

Supplement: S6 Fig — (A) Expression (cells) and secretion (media) of Hcp from wild-type (WT; T6SS+) A. jandaei DSM 7311, a T6SS− mutant strain (∆tssB), and a vgrG4 deletion strain (∆vgrG4) grown for 3 h at 30 °C in media containing 1% (wt/vol) NaCl (LB). RNA polymerase beta subunit (RpoB) was used as a loading and lysis control. Results from a representative experiment out of at least three independent experiments are shown. (B) Viability counts (colony forming units; CFU) of E. coli MG1655 prey strains before (0 h) and after (4 h) co-incubation with the indicated A. jandaei DSM 7311 attacker strains on LB plates at 30 °C. The statistical significance between samples at the 4-h time point was calculated using an unpaired, two-tailed Student t-test on log-transformed data; WT, wild-type; DL, the assay’s detection limit. Data are shown as the mean ± SD; n = 3. The data shown are a representative experiment out of at least three independent experiments. The data underlying this figure can be found in S6 Data. (TIF) [file pbio.3003053.s008.tif]

Fig. 2F

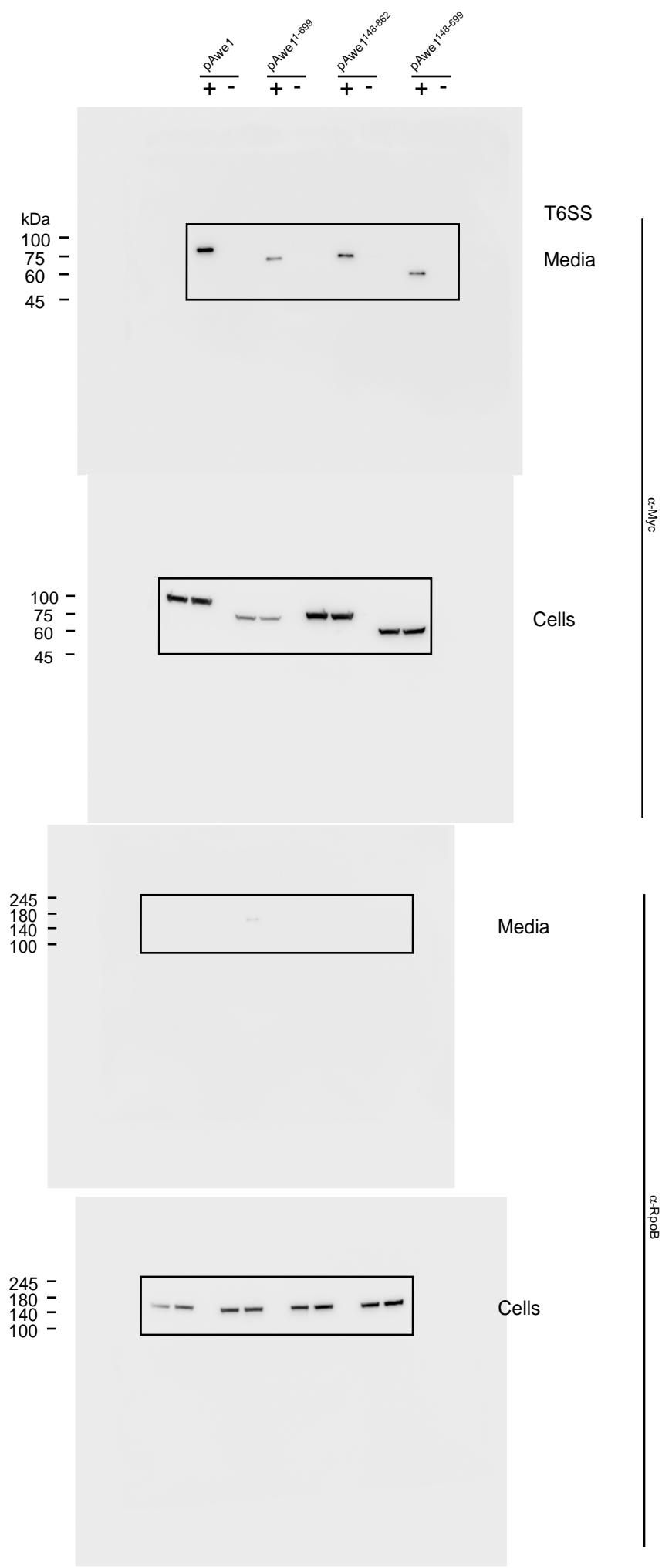

Fig. 2G

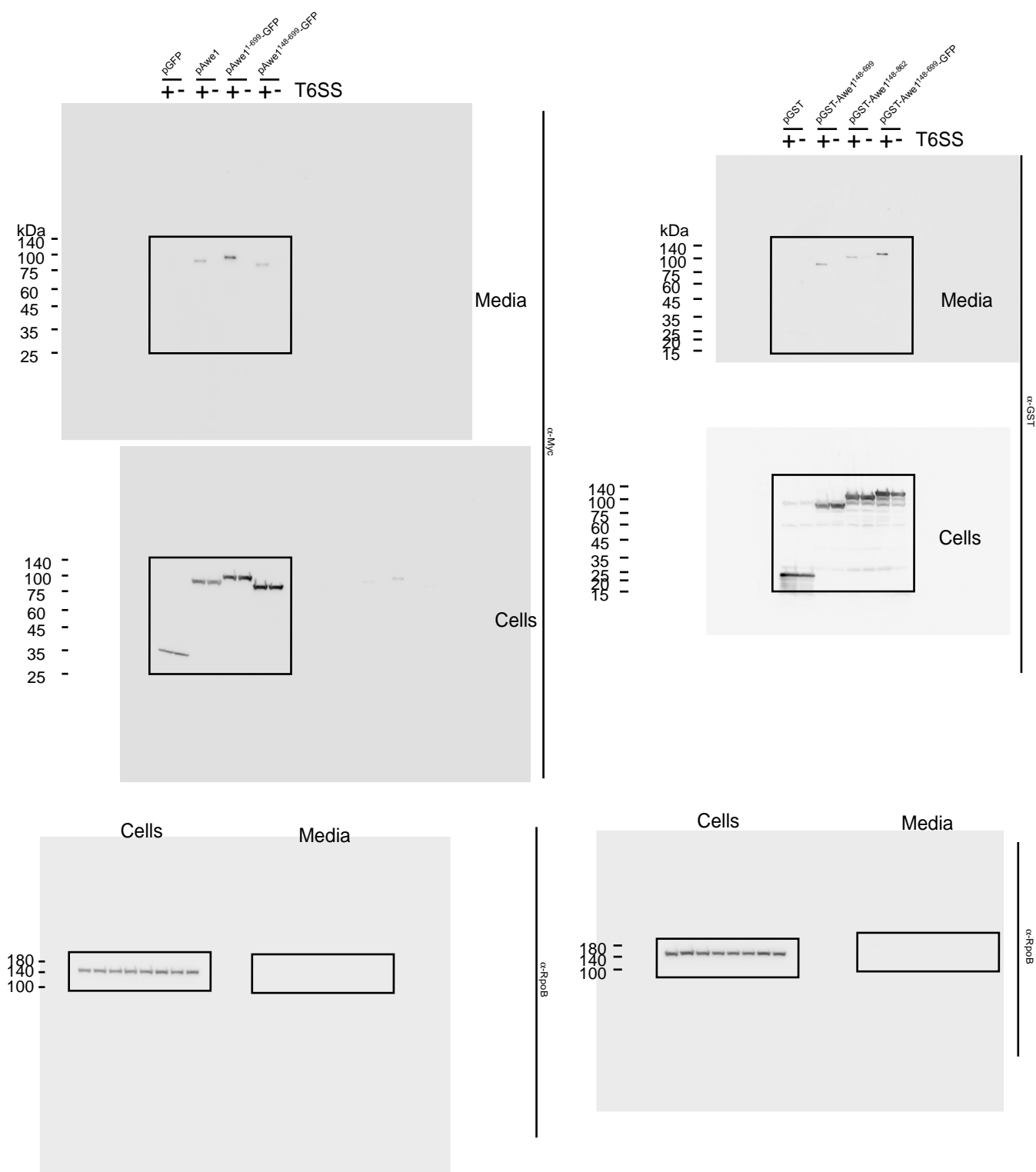

**Fig. 4B**

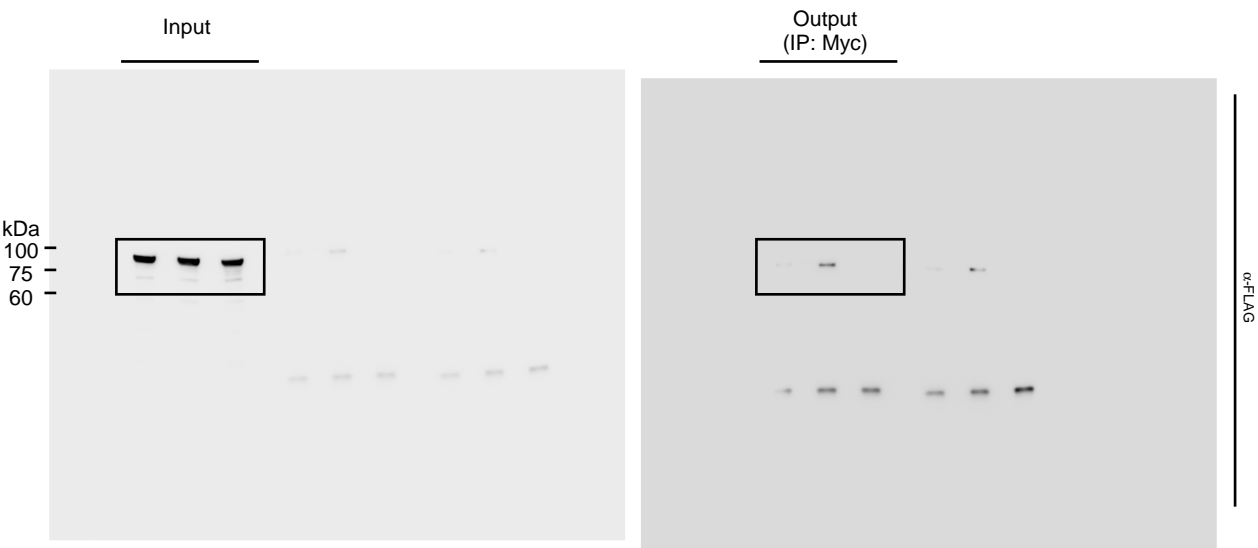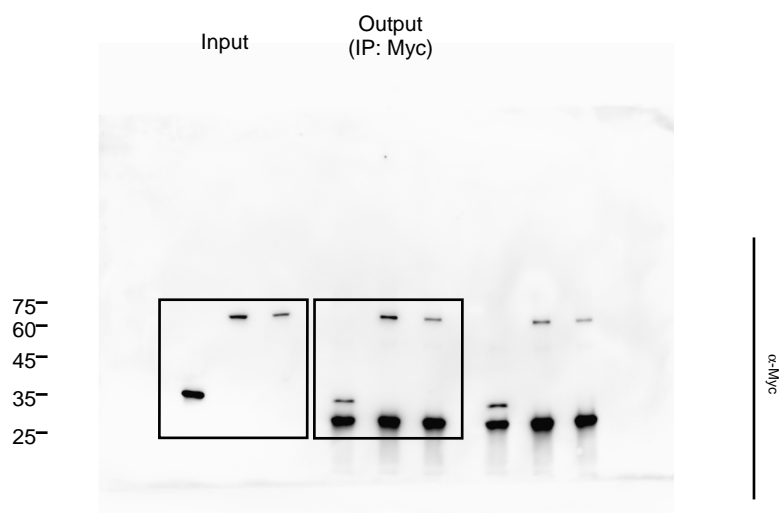

Fig. 4D

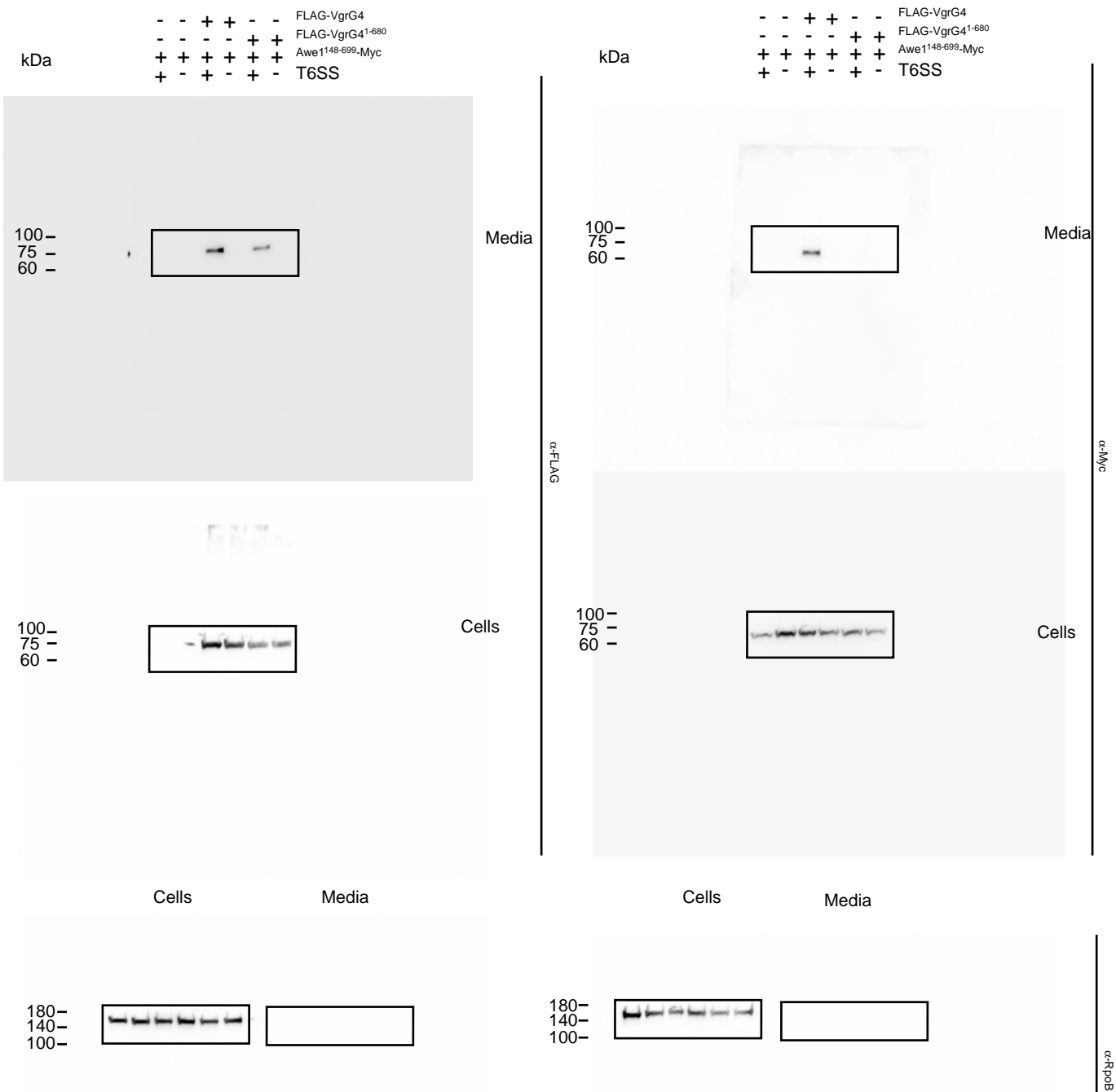

S2B Fig

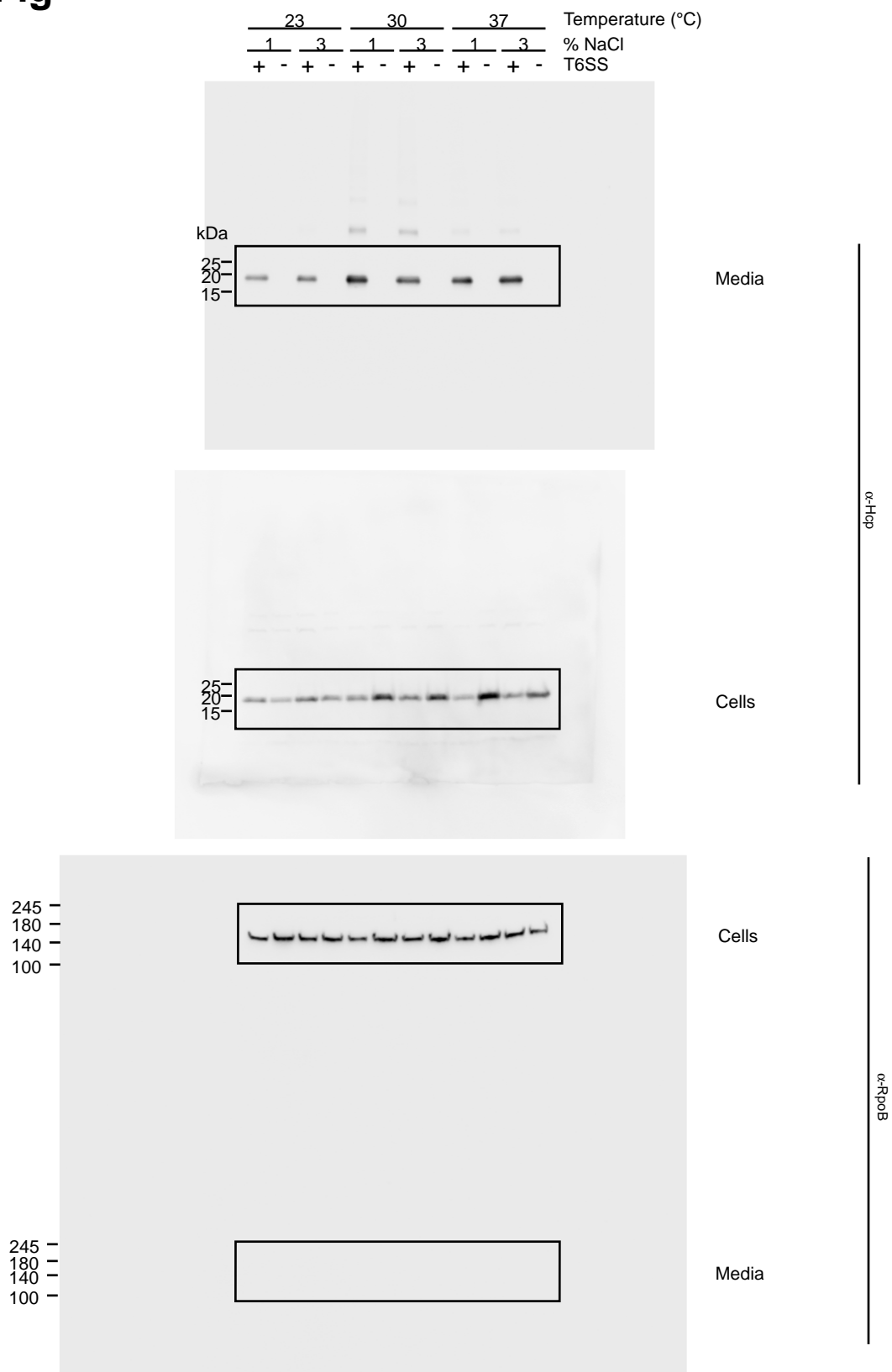

S2C Fig

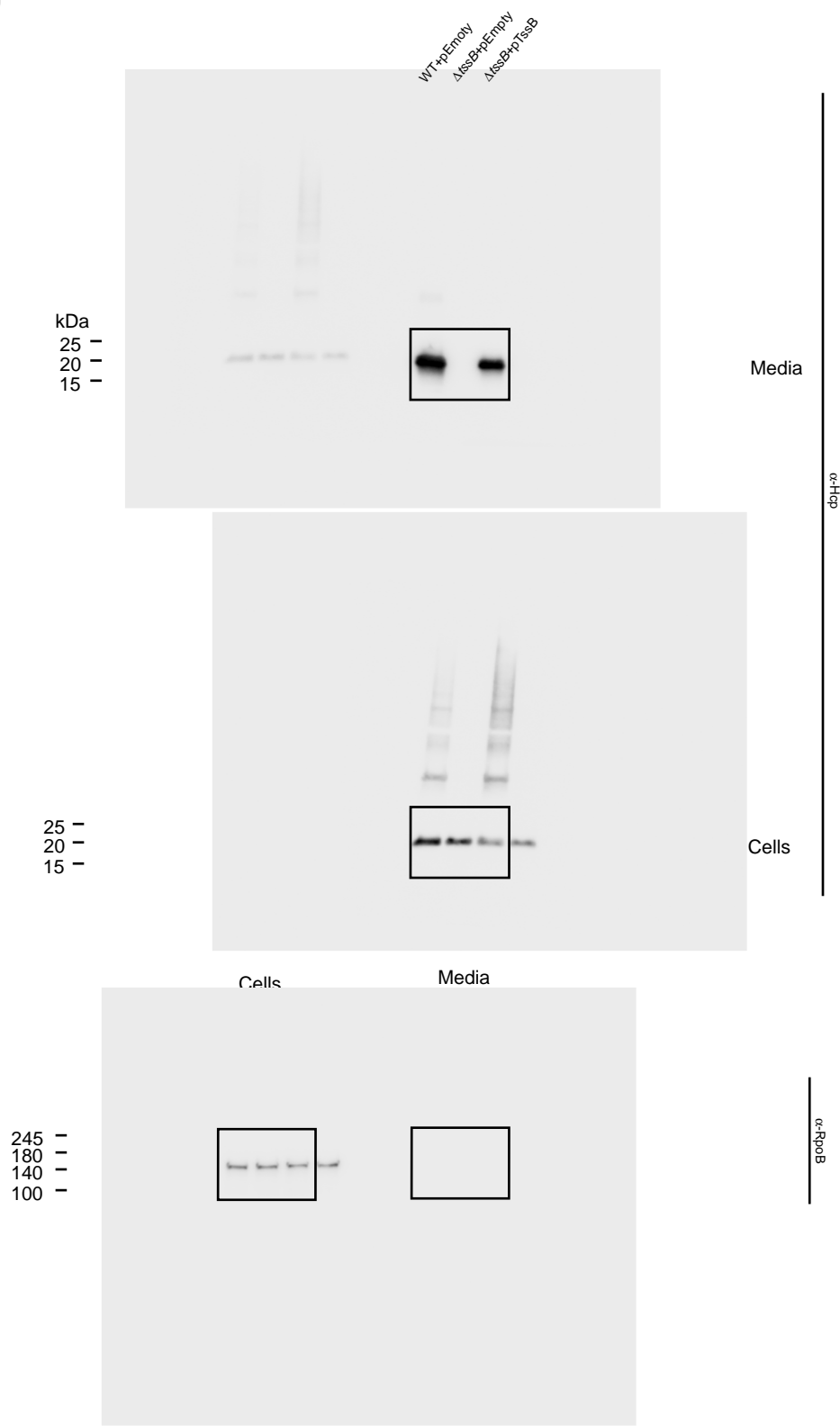

**S5 Fig**

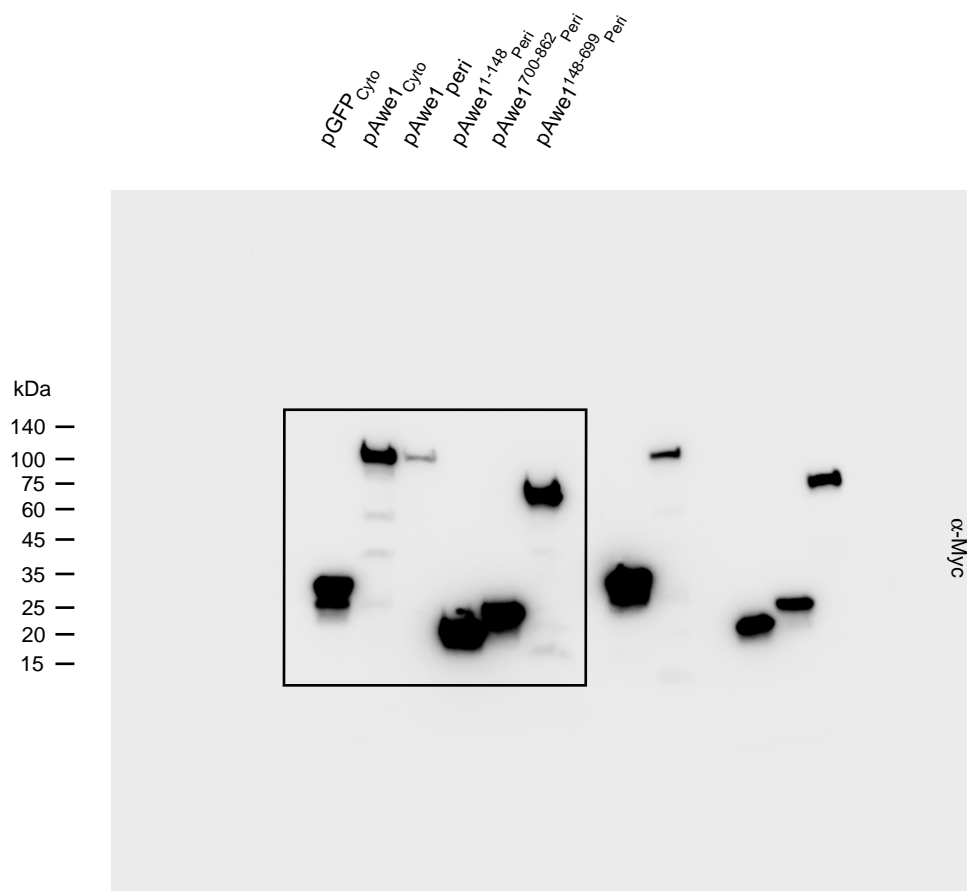

S6A Fig

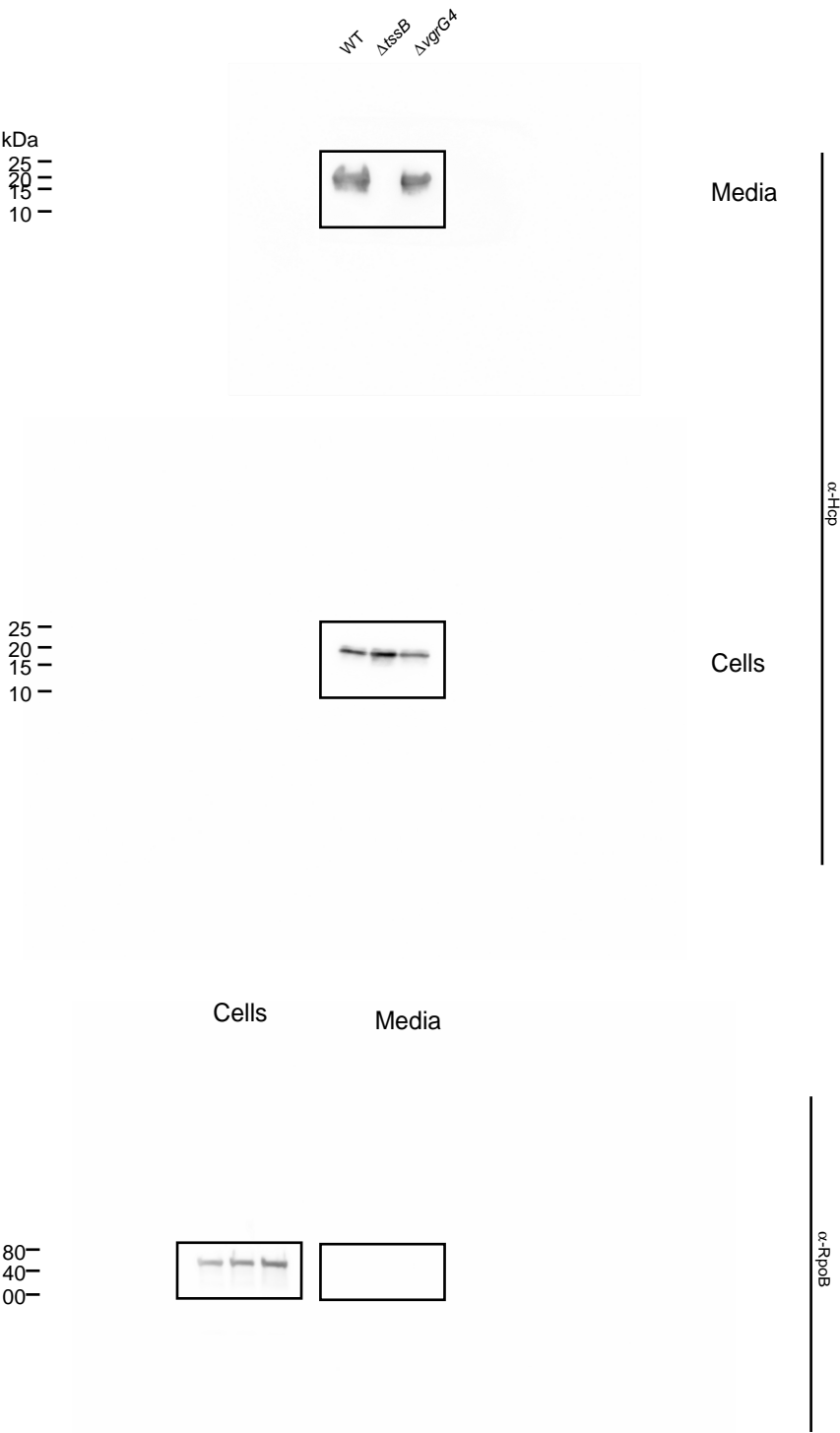

Supplement: S1 Raw Images — (PDF) [file pbio.3003053.s025.pdf]
