# Supplementary material for: A new class of type VI secretion system effectors can carry two toxic domains and are recognized through the WHIX motif for export
Source: PLoS Biol. 2025 Mar 17;23(3):e3003053. doi: 10.1371/journal.pbio.3003053 (PMC12135965; doi:10.1371/journal.pbio.3003053)
Supplement: Attachment — Submitted filename: PBP_response_to_reviewers_3_auresp_3.pdf [file pbio.3003053.s029.pdf]

Point-by-point response to reviewers' comments

Reviewer #1:

In this work, the authors have identified a new family of type VI secretion system effectors consisting of a VgrG-associating domain that can have additional functional domains fused to the N- or C-terminal of the domain. Although the results are interesting, there is a heavy reliance on computational predictions that are not fully experimentally confirmed. Additionally, the title using the phrase "double-edged sword" is very misleading.

We thank the reviewer for the valuable suggestions.

Figure 2B - showing this figure is not particularly meaningful... T6SS seems to be active under all conditions tested. Considering there is variation amount of protein in the control between samples, it's not like one can make a definitive regarding when T6SS is "most" active (lines 177-179).

Following the reviewer's comment, we replaced the secretion assay and competition assay in Fig. 2 (panels B and C in the previous figure version) with a competition assay showing the T6SS-mediated toxicity under different temperature and salinity conditions (New Fig 2B). We moved the secretion assay results to S2A Fig. Furthermore, we revised the text and removed the claim that the T6SS is "most" active under one specific condition (lines 146-149): "… although toxicity was apparent under all tested temperature and salinity conditions, it was significantly more pronounced in low salt media (LB; 1% [wt/vol] NaCl) compared to high salt media (MLB; 3% [wt/vol] NaCl) (Fig 2B). Based on these findings, we performed subsequent assays in media containing 1% [wt/vol] NaCl (LB) at 30°C".

The claim that "Aeromonas jandaei T6SS secretes four antibacterial effectors", is a bit weak. TseI, Tle1 and Awe1 are just hypothetical effectors based on "computational identification" and assumed activity. Only Awe1 and the 4th effector are actually demonstrated to be antibacterial effectors in this study. Also, how confident are the authors that there are not additional unidentified effectors?

Following the reviewer's comment, we now include self-competition assays demonstrating that TseI, Tle1, and DUF3289, together with their downstream genes, are antibacterial T6SS effector and immunity pairs (S3 Fig; lines 173-181). In addition, please note that the claim that these are T6SS effectors is not merely computational, as they were clearly identified in a comparative proteomics analysis as secreted by the T6SS (Fig. 2C).

Although we cannot exclude the presence of additional effectors, the deletion of these four effectors resulted in a strain that was no longer toxic to *E. coli* prey during competition (Fig. 4C). We revised the title of the relevant result section to "Aeromonas jandaei T6SS secretes *at least* four antibacterial effectors*"*.

Lines 280-281: The term "double-edged sword" generally refers to something that has both positive and negative effects (i.e. something that is both advantageous and disadvantageous).

Using this term strongly implies that that WHIX are somehow self-harming... which is not really true. The WHIX domain, while literally having two "edges" is not really a "double-edged sword".

We replaced the term “double-edged sword effectors” with the term “double-blade effectors” whenever appropriate in the text. We also revised the title of the manuscript to read “WHIX is a T6SS secretion domain found in polymorphic double-blade effectors”.

Figure 5 - how do you know that deleting VgrG4 doesn't disrupt T6SS activity? T6SS in other organisms have been demonstrated to have an essential VgrG, even when there are multiple VgrGs in play (e.g. V. cholerae). The control showing hcp secretion or even just E. coli killing in the ΔVgrG4 strain should be shown. Also, it would be nice to see the the WHIX construct (including the version with heterologous GST/GFP domains) being secreted with full-length VgrG4 but not with the truncated VgrG4.

We agree with the reviewer. We now include a control competition assay result demonstrating that a Δ*vgrG4* strain maintains a functional T6SS as it is toxic to *E. coli* prey. Furthermore, we show that Hcp is still secreted in this mutant strain (S6 Fig; lines 292-293).

In addition, we now demonstrate that secretion of the Awe1 WHIX domain is dependent on the presence of the full-length VgrG4, since it is no longer secreted when the C-terminal truncated VgrG4 version is expressed (Fig. 5D; lines 297-299).

Lines 360-362: VgrG4 being secreted in a T6SS-dependent manner (5C) does not imply anything about Awe1. This is "begging the question". The authors are presupposing that Awe1 must be interacting with VgrG4 without ever actually demonstrating the interaction and using that presupposition to conclude that Awe1 is associated with VgrG4.

Following the reviewer’s comment, we now demonstrate a direct interaction between VgrG4 and the Awe1 WHIX domain using co-immunoprecipitation, and we show that this interaction is dependent on the C-terminal end of VgrG4 (Fig. 5B; lines 283-287).

Line 422-423: The authors hypothesize that carrying two toxic domains reduces the risk of resistance developing, however in their experiments, they explicitly demonstrate that only a single immunity protein AwiD is sufficient to confer protection against the entire polymorphic Awe1 effector under native conditions. Some discussion of this apparent contradiction is probably warranted.

We note that the reviewer’s comment is correct only when *A. jandaei* is the prey strain (i.e., self-competition), as shown in Fig. 4B. However, we demonstrate in the revised Fig. 4C that against *E. coli* prey, both Awe1 toxic domains are functional and that only co-expression of AwiU and AwiD provides protection against the full-length effector (lines 261-267). We now mention in the discussion that the N-terminal toxic domain may target a component that is different in *Aeromonas*, and we also include in the discussion an alternative possibility in which the two toxic domains may be functional under different conditions, thus expanding the range of conditions in which the double-edged effector is toxic to the prey (lines 336-341): “It is possible that the N-terminal toxic domain targets a peptidoglycan component that differs between *Aeromonas* and other bacteria. Alternatively, two toxic domains may allow the effector to function under a wider range of conditions. For example, if the N-terminal and C-terminal toxic

domains are active in the prey cell under different salinity or temperature conditions. Such conditional effector toxicity was previously reported in effectors of *Pseudomonas aeruginosa*."

Line 443-445: This speculation seems to come out of nowhere. What reason do the authors have to think that WHIX could play an active role?

The rationale leading to this speculation is laid out in the preceding paragraph. Because WHIX domains appear to specifically associate with toxic domains that target peptidoglycan, it is possible that they play a role within the target cell and not just in association with the T6SS tube-spike for secretion. Otherwise, we would expect to find WHIX domains fused to toxic domains with diverse activities, as previously reported for other T6SS secretion domains (e.g., MIX, PIX, and RIX).

I wasn't able to open Supporting File S2

We uploaded the file again (now S3 File). To open the ".clans" files (S2 and S3 files), the CLANS application, available from the MPI Bioinformatics Toolkit (https://toolkit.tuebingen.mpg.de/tools/clans), should be downloaded.

Reviewer #2:

The manuscript entitled "WHIX is a T6SS secretion domain found in polymorphic double-edged sword effectors" authored by Mushka et. al. details the identification of a domain which demonstrates the ability to carry two distinct effector proteins which function by targeting cell's periplasmic regions and peptidoglycan. While working with Aeromonas jandaei, the authors identified a T6SS effector protein (Awe1) which carries two toxic domains each with their own immunity proteins (AwiD and AwiU). The N-terminal toxic domain, though not effective against Aeromonas, was toxic to E. coli prey. A potential mechanism of secretion for WHIX-containing effector was suggested to be associated with the C-terminal tail of VgrG4.

Overall, the manuscript is well written with a logical progression. Multiple methods were employed ranging from meta-sequence analysis, proteomics, protein structure, and more classic mutation-based experimentation with appropriate controls. However, the many weaknesses were noted and are summarized below.

We thank the reviewer for the valuable suggestions.

Comments

1. Discovery of fourth effector protein (WP_042032936.1) with DUF3289 in the result section is interesting and probably should be part of the main manuscript [1-3] rather than the supplemental figures. It is also recommended to remove the results depicted in figure 5 and move supplemental figures S3 into the main body of the paper. Briefly mentioning these findings at the end of the results section in figure 5 leaves the reader disoriented from the main takeaways of the rest of the manuscript, lessens the impact of the other findings, and brings up more questions than it does answers.

We thank the reviewer for these suggestions. The manuscript focuses on Awe1 and the WHIX domain. Therefore, we prefer to keep the data on the other effectors identified in *A. jandaei* as supplemental material. Please note that we have added new results demonstrating the activity of the other effectors identified in *A. jandaei*, TseI and Tle1, during interbacterial competition (S3 Fig.).

Respectfully, we intend to keep the results shown in Fig. 5 as part of the manuscript. They have now been expanded with additional experiments and data on the interaction between Awe1 and VgrG4, and they are relevant to the mechanism of WHIX secretion.

2. Throughout the manuscript, the statistical significance test which was chosen to analyze the data was an unpaired, two-tailed, student T-test. This is not the appropriate test to use in any of the cases here, as multiple groups were being compared to each other (i.e., figure 5B, in which case a one-way ANOVA is called for) or in some cases, multiple variables across multiple groups (i.e., figure 4B in which case a two-way ANOVA is called for). Analyzing multiple groups using a student T-test, which is designed for comparisons between two groups alone, compounds the chances of making a type-1 error leading one to underestimate the error in the experiment. This can potentially lead to finding significance in data where there is none.

Statistical analyses of all competition assays have been revised. Details are found in the respective figure legends.

3. Line 356-357 draws the conclusion "these results indicate that VgrG4 is required for Awe1 delivery", which is an overarching claim for the experiment that was performed. Deleting vgrG4 may have just abolished the efficacy of the entire T6SS and therefore the prey was not intoxicated. This experiment needs to be repeated with a built-in control to prove the continued function of the T6SS after VgrG4 deletion to make the claim this protein is required for Awe1-specific secretion.

We agree with the reviewer. We now include a control competition assay result demonstrating that a Δ*vgrG4* strain maintains a functional T6SS as it is toxic to *E. coli* prey and retains secretion of Hcp (S6 Fig; lines 292-293). We also show in a co-immunoprecipitation assay that VgrG4 binds the Awe1 WHIX domain (Fig. 5B; lines 283-285) and that the C-terminus of VgrG4 is required for WHIX secretion (Fig. 5D; lines 297-299).

4. In the introduction, a background of the T6SS and its antibacterial use is given. However, no mention is made of the T6SS and its host-modulatory effects, a well-established phenomenon [4]. A mention of this in the introductory material would be useful, as in line 182 other roles besides "interbacterial competition" is inferred.

We revised the Introduction to include information on anti-eukaryotic activities (lines 37-39).

5. In figures 1A and 1E, the N-terminus and C-terminus should be visually indicated. This would help avoid any confusion potentially felt by the reader, especially one less familiar with standard amino acid sequence annotations.

We revised Fig. 1A and 1E and denoted the N- and C-terminal domains.

6. In line 116, the 99 amino acid extension on the N-terminus of the subclass II WHIX-containing effectors is first mentioned. It is assumed this extension was the green triangle indicated in figure 1E as "immunity". It is suggested indicating this N-terminus region which divides subclass II WHIX-containing protein from subclass I with some sort of bracket and label in figure 1E to clearly indicate to the reader what is being referred to.

Each arrow in schematic representations denotes a single gene. The green arrow in Fig. 1E is an immunity gene, as denoted. The N-terminal extension was not clearly marked in the original figure, and is now marked with color in the revised figure panel, as suggested by the reviewer.

7. Lines 144-148 detail the T6SS gene cluster found in A. jandaei and mentions the existence of two 'auxiliary modules' each with its own effector protein. In figure 2A, a third module is depicted with a putative effector labeled "DUF3289". This effector is addressed later on in the manuscript, but at this point appeared to be left untouched. It is suggested adding a mention of it here, with further details later on. Same thing in line 180. Three effectors are mentioned but figure 2A shows four effectors, so this can be confusing.

Following the reviewer's suggestion, we now mention this effector when describing Fig. 2A (lines 131-132).

8. Lines 230-231 describe the results of modeling the binding of immunity proteins AwiU and AwiD to Awe1. Does the prediction that the immunity proteins bind to the N- and C-terminal domains of the Awe1 effector have anything to do with the fact that, genomically they are located directly up and downstream of the awe1 gene? seems obvious that feeding the awe1 sequence along with the sequences of the immunity proteins into alphafold3, the program will predict the sequences on either end of awe1 be attached to those same ends after translation? Or is the input for this model three separate sequences and still the predicted binding in the same orientation they exist on the genome? It is not clear in the paper.

The AlphaFold prediction of the complex is made using three separate protein sequences. This can also be seen in the PDB file provided in S6 File. To clarify this point, we revised the following sentences in the text. Lines 189-192: "Furthermore, when the Awe1 structure was predicted *in a heterotrimer* together with the putative immunity proteins encoded upstream (AwiU) and downstream (AwiD) of Awe1 (Fig 2A), we found that these immunity proteins are expected to bind the N-terminal and C-terminal domains of Awe1, respectively."

9. In the description for figure 3D in line 265, its mentioned that 'full-length Awe1' secretion was monitored. In reality, an anti-Myc-tagged version of full-length Awe1 secretion expressed from a plasmid was actually measured after the deletion of native awe1 in either T6SS+ or T6SS- strains. This wasn't clear until reading the figure legend and this detail should be mentioned in the body of the text.

The relevant text was revised as suggested. Lines 209-213: "To determine whether the Awe1 WHIX domain is sufficient to mediate T6SS-dependent secretion, we monitored the secretion of plasmid-expressed full-length Awe1 *fused to a C-terminal Myc tag*, or its N- and C-terminal truncated forms (Fig 3D) from *A. jandaei* in which we deleted the genomic awe1 (Δawe1; T6SS+) or from a derivative in which we inactivated the T6SS (Δawe1/ΔtssB; T6SS^{-})."

10. The conclusion in line 344-345 should include a mention of the fact that the N-terminal domain's toxicity may be specifically directed at other species, for example E. coli.

We thank the reviewer for this suggestion. We think that this distinction is more appropriate in the Discussion section than in the Results section. This is mentioned in the Discussion in lines 331-341: "We hypothesize that WHIX domains' ability to carry two fused toxic domains reduces the risk of resistance developing in prey strains [73], as well as expands the potential target range of a single effector. Indeed, we find that the N-terminal toxic domain of Awe1 does not intoxicate Aeromonas in self-competition, yet it is functional against *E. coli* prey in which both the downstream-encoded immunity protein and the upstream-encoded immunity protein are required to antagonize Awe1-mediated toxicity. Alternatively, two toxic domains may allow the effector to function under a wider range of conditions. For example, if the N-terminal and C-terminal toxic domains are active in the prey cell under different salinity or temperature conditions. Such conditional effector toxicity was previously reported in effectors of *Pseudomonas aeruginosa*."

11. A brief description of VgrG proteins in line 348 should be added as a reminder to readers, so it is clear the role these proteins play in the T6SS before getting into the results of this section.

We added a sentence at the beginning of the paragraph and revised the sentence mentioned above according to the reviewer's suggestion. Lines 277-280: "*Many T6SS cargo effectors were shown to be loaded onto a secreted T6SS tube-spike core component encoded next to them on the genome.* Therefore, we hypothesized that Awe1 is loaded onto the upstream-encoded *secreted spike component* VgrG4 (WP_198493475.1) for delivery via the T6SS."

12. In line 350, the AlphaFold structure of WHIX is cited as a justification for the hypothesis that Awe1 WHIX domain is loaded onto the C-terminal tail of VgrG4. The 'deep cavity' in this domain mentioned in earlier results is specifically pointed to in the discussion section but it needs to be mentioned here in the results section as to why WHIX may be binding to the C-terminal domain of VgrG4.

We revised the sentence as suggested by the reviewer. Lines 280-283: "In agreement with our findings that the Awe1 WHIX domain is sufficient for T6SS-dependent secretion, an AlphaFold structure prediction implies that Awe1 is loaded onto VgrG4; *the predicted structure suggests that the deep cavity of the WHIX domain caps the C-terminal tail of VgrG4*."

13. Are there any plans for future work to be done on the mechanism of action against peptidoglycan? It is not mentioned as a future direction in the discussion section, which looks like an oversight because the putative toxicity of these WHIX domains to peptidoglycan specifically is mentioned throughout the paper and peptidoglycan as a target is even given as a final conclusion of the whole paper at the end in lines 455-457.

We added the following sentence at the end of the Discussion section.

Line 358-260: "In future work, we plan to investigate the mechanisms of action and the target of newly identified toxic domains fused to WHIX."

14. The second to last paragraph in lines 446-452 is just a restatement of results already given in the results section. More needs to be said in this paragraph about the implications and these results, and speculations for future work it spurs, and its impact on the field.

The following sentences were added to expand the discussion in this paragraph.

Lines 369-376: "Previous works investigating the T6SS effector repertoires of Aeromonas strains focused only on effectors encoded within auxiliary modules containing secreted tube-spike components [75,76]. Therefore, it will be interesting to determine whether other *Aeromonas* strains, besides *A. jandaei*, also carry orphan T6SS effectors. In addition, analyses of T6SS effector repertoires in other bacteria, such as *Vibrio parahaemolyticus* [77] and *Vibrio coralliilyticus* [78] revealed core and accessory T6SS effector repertoires. Once additional information on orphan T6SS effectors is available in *Aeromonas*, a similar analysis can be performed on their T6SS effector repertoires as well."

References

1. Jana, B., et al., A modular effector with a DNase domain and a marker for T6SS substrates. Nat Commun, 2019. 10(1): p. 3595.
2. Fitzsimons, T.C., et al., Identification of Novel Acinetobacter baumannii Type VI Secretion System Antibacterial Effector and Immunity Pairs. Infect Immun, 2018. 86(8).
3. Li, D.Y., et al., Identification and Characterization of EvpQ, a Novel T6SS Effector Encoded on a Mobile Genetic Element in. Front Microbiol, 2021. 12: p. 643498.
4. Singh, R.P. and K. Kumari, Bacterial type VI secretion system (T6SS): an evolved molecular weapon with diverse functionality. Biotechnol Lett, 2023. 45(3): p. 309-331.

Reviewer #3:

Fridman and coworkers aim to shed light into a new domain, WHIX, found in T6SS effectors. Effectors containing this domain may have one or two toxic domains, and there seems to be distinct immunity proteins. Authors postulate that WHIX acts as a cargo domain. Additionally, authors provide an initial characterization of Aermonas jandaei T6SS system.

General comment.
It adds to the T6SS field the characterization of a putative new cargo domain although the evidence is not solid enough yet to make the point that a new family of effectors has been identified. What authors have described is common to many effectors across (toxic domains with immunity protiens around and/or closely associated with a VgrG protein). No characterization of the target and mode of action of the effector is presented and much of the structural work relies on in silico predictions with limited experimental validation. The section on Aermonas general characterization of the T6SS while well done distracts from the message of the paper and in fact does not add much to the overall notion of the work. Hope that the comments will help the authors to strengthen the case they are trying to make.

We thank the reviewer for the valuable suggestions. As detailed below, we added experimental data and computational analyses to strengthen our conclusions and alleviate concerns raised by the reviewer. Notably, although we agree with the comment that the section characterizing the T6SS in *A. jandaei* deviates from the main message of the work, it is nonetheless an essential part that establishes the conditions and tools that are used in subsequent assays to investigate the effector Awe1 and its WHIX domain.

Major comments.
1. To further strengthen the analysis in Fig 1B, authors should analyze the distribution of the the toxic domains in the putative WHIX protein identified: (i) which is the prevalence of one and two putative toxic domains? which is the diversity of toxic domains? is there a topic of toxic domain over represented?

Following the reviewer's suggestions, we expanded the analysis of domains fused to WHIX. The diversity of the toxic domains fused to the C-terminal and N-terminal ends of WHIX is now shown in Tables 1 and 2 and in S1 Fig. Further detailed domains identified in each WHIX-containing protein are provided in S1 dataset.

The prevalence of the double-blade WHIX effectors (i.e., subclass II) is ~16% of the unique protein accession numbers (line 96). We now also denote the different subclasses by colors in Fig. 1E.

2. Fig 2B. To rigorously make the case, authors would need to quantify the 3 immunoblots, and add data related to differences of transcription between temperature and NaCl content.

Following the reviewer's comment, this Results section was revised, and the claim that specific temperature conditions are ideal for T6SS activity has been retracted. The revision includes additional experimental data in which we quantified the T6SS-mediated antibacterial toxicity in different temperature and salinity conditions (Fig. 2B; the Hcp secretion assay is now S2A Fig).

We now conclude, based on the competition and Hcp secretion data, that T6SS activity is better in low salinity media, with no claim to a specific ideal temperature. Lines 140-151: "The effectors that we computationally identified in *A. jandaei* are encoded next to putative immunity genes (Fig 2A). Therefore, we hypothesized that this T6SS plays a role in interbacterial competition. To test this hypothesis, we used the wild-type *A. jandaei* and its T6SS⁻ mutant strain (Δ*tssB*) as attackers in competition against *Escherichia coli* MG1655 prey. Both wild-type and the *tssB*-complemented mutant attacker strains, but not the T6SS⁻ mutant containing an empty plasmid, killed the *E. coli* prey during a 4-hour co-incubation, as evidenced by a drop in prey viability; although toxicity was apparent under all tested temperature and salinity conditions, it was significantly more pronounced in low salt media (LB; 1% [wt/vol] NaCl) compared to high salt media (MLB; 3% [wt/vol] NaCl) (Fig 2B). Based on these findings, we decided to perform subsequent assays in media containing 1% [wt/vol] NaCl (LB) at 30°C. Taken together, our results indicate that the T6SS in *A. jandaei* is active under a wide range of growth conditions in which it mediates antibacterial toxicity."

3. Authors may need to revise the statistical analysis of the manuscript. In most cases (Fig 2, 4 and 5) it is more appropriate a two-side ANPOVA with multiple comparisons correction.

Statistical analyses of all competition assays have been revised. Details are found in the respective figure legends.

4. The binding of the immunity proteins to Awe1 can be tested experimentally by, for example, bacteria two-hybrid experiments. Additionally, authors need to establish whether each of the immunity proteins bind to the cognate toxic domain.

As suggested by the reviewer, we now provide a BACTH result demonstrating that the predicted immunity proteins specifically bind to their cognate Awe1 toxic domain (Fig. 3C; lines 192-194).

5. Fig 3D. Authors need to quantify the 3 immunoblots. The results that there is a reduction in the levels of secreted effector in all the truncated forms, particularly noticeable in the one lacking the N-terminal domain. If this is so, this will impact the killing experiments presented later on in the manuscript.

Although it is possible that reduced secretion levels of the truncated effector forms compared to the full-length effector affect the potency of the T6SS-mediated killing, it should not impact the conclusions drawn from the competition assay results shown in Fig. 4B-C. We analyze the effect of the truncated versions in a binary format – toxic or not toxic during competition, and we do not compare their toxicity to that of the full-length effector.

6. Which is the Hcp responsible for the secretion of Awe1? Is there any promiscuity in terms of the VgrG that can secrete Awe1? It will be crucial to demonstrate whether indeed WHIX domain interacts with VgrG and the specificity of interaction with the 14 C-terminal AAs. As noted before, bacteria two hyvrid epxerimets may be useful here coupled with secretion experiments.

Regarding Hcp – two of the Hcp proteins encoded by *A. jandaei* are identical (Hcp1 and Hcp3), and Hcp2 shares 98.26% identity with them. Therefore, it is not possible to determine which one is required specifically for the secretion of Awe1.

Regarding the VgrG responsible for Awe1 secretion – we now include a co-immunoprecipitation result showing that the WHIX domain of Awe1 binds VgrG4, and that this binding is dependent on the C-terminal tail of VgrG4 (Fig. 5B). Furthermore, in Fig. 5C we show that deletion of *vgrG4* abrogates Awe1-mediated toxicity during self-competition (while not abrogating overall T6SS activity; S6 Fig), and in Fig. 5D we show that the Awe1 WHIX domain is not secreted in the absence of the C-terminal end of VgrG4. Collectively, these results indicate that VgrG1-3 are unable to compensate for the loss of VgrG4 to mediate Awe1 secretion.

7. Fig 3E, as noted before authors need to quantify the 3 immunoblots. These constructs will also help authors to clarify the previous comment on secretion of the effector. Here it seems that the GST-WHIX-GFP chimeric protein is the best secreted.

Please note that we make no claims of comparisons between the different expression and secretion levels of the proteins shown in this figure. We only claim secretion by the T6SS. Therefore, quantification of the immunoblots will have no impact on our results and conclusions.

Furthermore, we do not think that ECL-based immunoblot quantification is reliable; therefore, we avoid using it to draw quantitative conclusions.

8. Authors need to establish the target and mode of action of Awe1, and assess whether each of the domains have a different function or they complement each other. This is essential to sustain the claim of a double sword effector. If both domains have the same target and mode of action then it will be challenging to justify the claim of the authors.

Our focus in this work is on WHIX being (1) a new T6SS secretion domain that defines a widespread family of polymorphic toxins, and (2) the first known example of a T6SS secretion domain able to carry two distinct toxic domains that function independently (double-blade effector). The latter is demonstrated by the toxicity mediated by each domain in the competition assays and the requirement of two cognate immunity proteins to antagonize it, as shown in Fig. 4. It is further supported by the finding that each domain has a different target range (i.e., the C-terminal toxic domain is detrimental to both *Aeromonas* and *E. coli* during competition, whereas the N-terminal domain is not toxic towards *Aeromonas*). Although we plan to investigate the activity of the different toxic domains in future work, we think that this is not required to sustain the claim of a double-blade effector.

Minor comments.

1. In Figure 1, a pictorial representation of the class I and II would help to better appreciate the differences.

As suggested by the reviewer, Fig. 1E now clarifies the differences between the WHIX subclasses.

2. Based on the proteomic analysis, which is teh connection between T6SS and motility iin Aeromonas? Is a T6SS mutant motile?

We thank the reviewer for raising this interesting question. Indeed, it has been reported in several bacteria that T6SS inactivation also affects the secretion or expression of Flagella components (https://doi.org/10.1099/mic.0.063495-0 and https://doi.org/10.1016/j.aquaculture.2023.739899), as implied from our proteomics data in *A. jandaei*. However, this topic is beyond the scope of the current manuscript, and we did not pursue it.

Reviewer #4:

In this manuscript, the authors identify a conserved domain (named WHIX) that is present in thousands of proteins encoded on genomes of T6SS positive bacteria. The authors show that this conserved domain can be extended on both the C- and N-terminus with additional protein domains that often represent putative effectors. In addition, periplasm targeted immunity proteins are often encoded next to such effectors. The authors then identify an active T6SS cluster in Aeromonas jandaei, which secretes an effector (Awe1) with this new domain in a T6SS dependent manner and can kill E. coli. Interestingly, this effector has both N- and C-terminal domains that are potentially toxic to target cells. The authors showed that those two domains can be removed without affecting secretion and even replaced with GST and GFP, which are then secreted in a T6SS-dependent manner. The effector as well as the two domains are toxic when expressed in the periplasm of E. coli but only one has a major impact on the outcome of competition assays. Finally, this novel effector requires a cognate VgrG (its last 14 aa) to get secreted and delivered into target cells. Overall, this manuscript is clearly written, the data is solid and well presented, there are appropriate controls available and thus the conclusions are justified. It is an interesting and important addition to our understanding of T6SS effector reservoir. I have no major comments and I congratulate the authors on their manuscript.

We thank the author for the kind assessment of our work.
